# Sensor Data-Driven Bearing Fault Diagnosis Based on Deep Convolutional Neural Networks and S-Transform

**DOI:** 10.3390/s19122750

**Published:** 2019-06-19

**Authors:** Guoqiang Li, Chao Deng, Jun Wu, Xuebing Xu, Xinyu Shao, Yuanhang Wang

**Affiliations:** 1 School of Mechanical Science and Engineering, Huazhong University of Science and Technology, Wuhan 430074, China; lgq1211@tom.com (G.L.); dengchao@hust.edu.cn (C.D.); shaoxy@hust.edu.cn (X.S.); 2 School of Naval Architecture and Ocean Engineering, Huazhong University of Science and Technology, Wuhan 430074, China; m13627290949@163.com; 3 China Electronic Product Reliability and Environmental Testing Research Institute, Guangzhou 510610, China; wangyuanhang@ceprei.com

**Keywords:** fault diagnosis, convolution neural networks, S-transform, sensor data, bearing

## Abstract

Accurate and timely bearing fault diagnosis is crucial to decrease the probability of unexpected failures of rotating machinery and improve the efficiency of its scheduled maintenance. Since convolutional neural networks (CNN) have poor feature extraction capability for sensor data with 1D format, CNN combined with signal processing algorithm is often adopted for fault diagnosis. This increases manual conversion work and expertise dependence while reducing the feasibility and robustness of the corresponding fault diagnosis method. In this paper, a novel sensor data-driven fault diagnosis method is proposed by fusing S-transform (ST) algorithm and CNN, namely ST-CNN. First of all, a ST layer is designed based on S-transform algorithm. In the ST layer, sensor data is automatically converted into 2D time-frequency matrix without manual conversion work. Then, a new ST-CNN model is constructed, and the time-frequency coefficient matrixes are inputted into the constructed ST-CNN model. After the training process of the ST-CNN model is completed, the classification layer such as softmax performs the fault diagnosis. Finally, the diagnosis performance of the proposed method is evaluated by using two public available datasets of bearings. The experimental results show that the proposed method performs the higher and more robust diagnosis performance than other existing methods.

## 1. Introduction

Bearings are an extremely critical component in rotating machinery to reduce the friction between the moving parts and provide continuous effective support for the rotary axis. According to the investigation of rotating machinery failures, more than 45% of the machinery breakdown is caused by the bearing fault [1]. Thus, the bearing fault diagnosis has an enormous impact on maximizing the production efficiency of machinery, minimizing machinery downtime and the maintenance cost [2]. As a result, the bearing fault diagnosis has attracted considerable attentions.

In general, a typical bearing fault diagnosis includes three main steps: Monitoring signal acquisition, feature extraction, and fault diagnosis. In the monitoring signal acquisition step, sensor data such as vibration signals, acoustic emission signals, motor current signals, and temperature signals have been widely used in the field of fault diagnosis [3,4,5]. In addition, these signals are often collected by sensors mounted on the machinery. In the feature extraction step, a common practice is to extract time domain features such as the root mean square, skewness, kurtosis coefficient and gap factor from collected signals using statistical methods [6,7,8,9,10,11]. To a certain extent, time domain features can effectively expose the fundamental differences under different conditions. In addition, frequency domain analysis methods [12,13] based on Fourier transform [14,15] are used to extract discernible frequency features from the collected signals. However, the monitoring signals of bearings obtained from practical industrial applications have non-linearity and non-stationary characteristics. The use of the statistical methods and frequency domain analysis methods to deal with the signals have their inherent limitations. To address this problem, time-frequency domain analysis methods such as wavelet transform (WT), empirical mode decomposition (EMD) and Hilbert–Huang transform (HHT) are introduced [16,17,18,19]. The time-frequency analysis methods decompose the collected signals into a set of time-frequency components, and these time-frequency components contain some fault features, which are useful for fault diagnosis [20,21,22]. In the fault diagnosis step, machine learning methods are the basis for effective fault diagnosis. For example, Yan et al. [23] proposed a novel fault diagnosis algorithm based on optimized support vector machines with multi-domain feature to achieve fault diagnosis of bearings. Zhao et al. [24] used the improved Euclidean weighted K-nearest neighbor (EW-KNN) classifier to monitor various health conditions of rolling bearings. Zhang et al. [25] introduced wavelet packet decomposition to improve EMD for time-frequency feature extraction. Singh et al. [26] presented a bearing fault diagnosis method based on the principles of EMD, envelope analysis and pseudo-fault signal. In this regard, the machine learning-based fault diagnosis method inevitably needs to rely on designed representative features and complex model tuning. However, the designed representative features such as wavelet coefficient features and intrinsic mode functions (IMFs) have their inherent limitations in extracting high-frequency components which have the obvious fault characteristics. In addition, strong background noise and other interference components of signals inevitably exist in collected monitoring signals under practical industrial environment. Moreover, traditional fault diagnosis methods based on machine learning are not always effective to eliminate the effect of strong background noise and other interference components of signals.

Deep learning (DL), as a new field of machine learning, provides an efficient way to automatically learn representative features from collected signals. Several common DL methods have been used in the field of fault diagnosis. For instance, Jia et al. [27] proposed a fault diagnosis model for rotating machinery based on deep neural networks (DNN). Sun et al. [28] proposed a fault classification model for induction motor fault classification based on sparse auto-encoder and DNN. However, fully-connected DNNs have limitations in solving more complex problems. The parameters of DNN are exponentially increasing when more layers are needed for data fitting. It leads to high computational complexity and possible overfitting problems.

Compared with the DNN-based method, a convolutional neural networks-based (CNN) [29,30] method is easier to train under the same available training set and computational resource. With the widespread application of CNN in fault diagnosis, CNN has shown the capability in extracting useful and robust features from monitoring signals. Ince et al. [31] proposed a 1D CNN-based approach that is directly applicable to the raw signal and achieved a more efficient fault detection system for real-time motor. However, the raw signals are interspersed with noise interference components, which increase the requirement of feature extraction capability of 1D CNN and increases the training cost of 1D CNN. Han et al. [32] developed the CNN-based model for gearbox fault diagnosis with constructed multi-level wavelet coefficients matrixes for reducing the interference of noise in vibration signal. However, common time-frequency analysis methods such as WT, short time Fourier transform (STFT), and HHT have the limitations when they are used to convert the signals into time-frequency coefficient matrixes for obtaining the sensitive fault components. One is that the strong correlation of the fault features extraction method-based on CNN with the quality of time-frequency information imposes big challenges in the noise interference background applications. The other is that the arduously obtained time-frequency information by manual work increases the complexity of the fault diagnosis method based on CNN.

In this paper, we propose a new sensor data-driven fault diagnosis method based on S-transform (ST) and CNN, namely ST-CNN. In order to obtain the appropriate inputs of CNN and enhance the quality of inputs, the ST is used to obtain the time-frequency matrix from sensor data. Several researchers have presented their investigations using ST and generalized ST to obtain time-frequency representative of signals for bearing fault detection [33,34]. Admittedly, the generalized ST can make up the poor energy concentration of ST in high frequency domain. However, in the proposed method, the ST is used to deal with sensor data for obtaining the useful time-frequency matrix due to it having poor energy concentration at the high frequency. In addition, its frequency-dependent window function produces higher frequency resolution at lower frequencies; hence the low frequency fault components could be enhanced [35]. Based on the above, the ST is introduced into CNN as the ST layer. Through the ST layer, the time-frequency complex matrix with 2D format is automatically extracted from sensor data without manual conversion, and this matrix with 2D format is a suitable input for the CNN. By integrating CNN with the ST layer, the ST-CNN could directly use original sensor data to realize the bearing fault diagnosis.

The remainder of this paper is organized as follows. Section 2 presents the proposed ST-CNN architecture. In Section 3, the procedure of the proposed fault diagnosis method is explained in detail. In Section 4, two real experiments are conducted to evaluate the effectiveness of the proposed method, and the results and discussions are presented. The conclusions are given in Section 5.

## 2. Proposed ST-CNN Architecture

**Figure 1:**
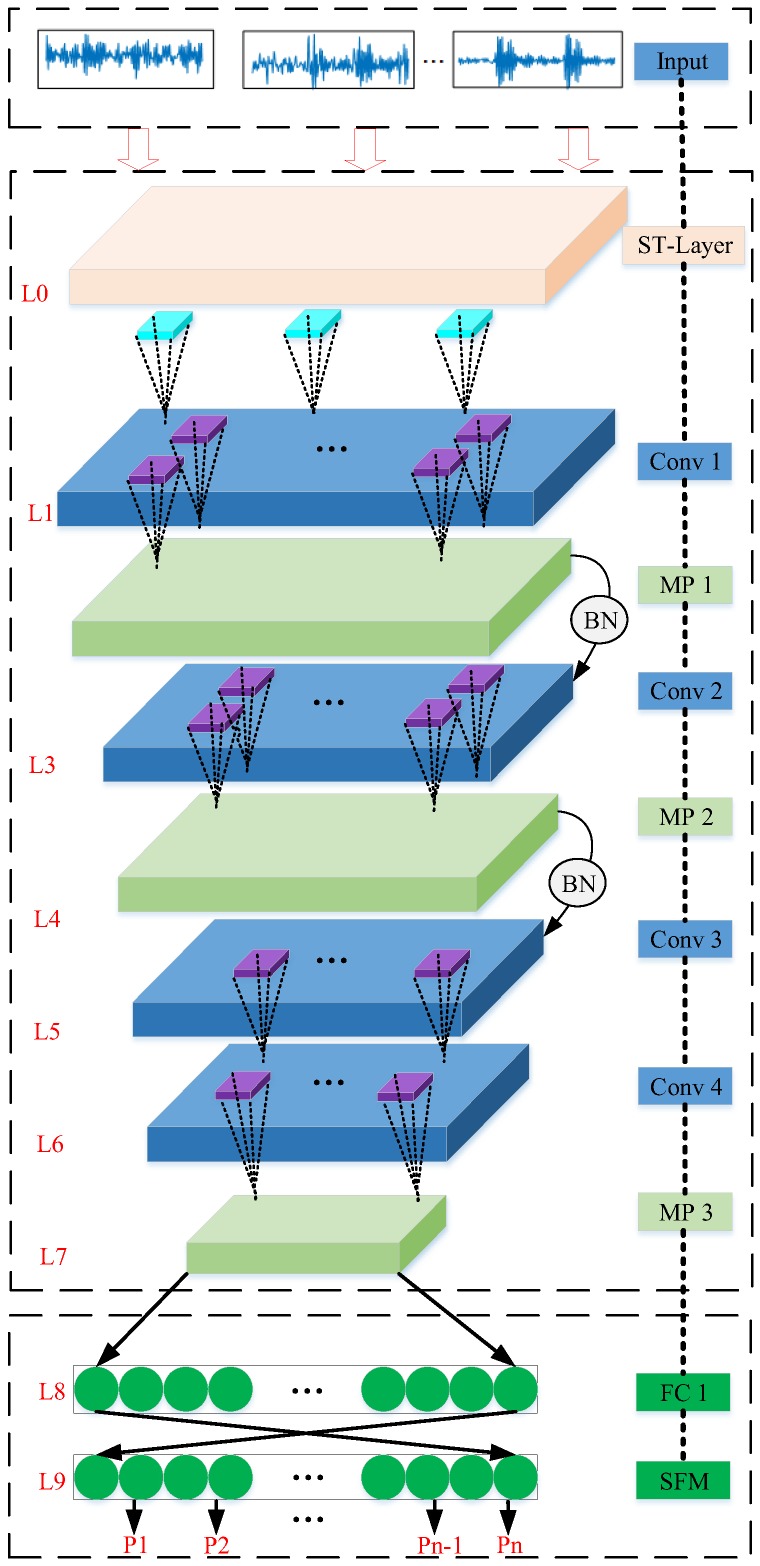
The architecture of the S-transform-convolutional neural networks (ST-CNN).

Admittedly, neurons generally have the function of information extraction and discrimination in the human brain. Considering this, an architecture based on artificial neurons is designed. The sensor data is inputted into the designed architecture. Through the calculation and transfer of artificial neurons, the representative features of sensor data could be obtained, and then the condition of sensor data also could be achieved. As shown in Figure 1, the architecture of the proposed ST-CNN consists of five most important components, that is, the ST layer, convolutional layer, pooling layer, fully-connected layer and classification layer. In this architecture, the ST algorithm is integrated into the ST-CNN as the ST layer, which feed the appropriate inputs to the first convolutional layer. Then, the convolutional layers (i.e., Conv 1, Conv 2, Conv 3, Conv 4) and the pooling layers (i.e., MP 1, MP 2, MP 3), are used to extract the representative features. The batch normalization (i.e., BN) is used between MP 1 with Conv 2 and MP 2 with Conv 3, respectively. The fully-connected layer (i.e., FC 1) is adopted to non-linearly fit the representative features extracted from MP 3. The classification layer (i.e., SFM) is used to output the probability that the testing signal sample belong to different fault types. The details of the operation of each layer is as follows:

### 2.1. ST Layer

Recently, the merits of CNNs have been represented in the field of extracting representative feature of images. However, CNNs are not compatible with 1D time-series sensor data. In contrast, CNNs are well suited to obtain the representative features from input with the 2D format. Due to the differences of sensor data between different operating conditions could be reflected in time-frequency data, researchers have investigated the fault diagnosis based on the time-frequency analysis method (such as WT and STFT) and CNN. As the extension of the ideas of WT, ST is based on a moving and scalable localizing Gaussian window to obtain the desirable time-frequency feature, which is absent in WT [36]. In this regard, ST is used to deal with the sensor data to obtain the time-frequency matrix [37].

In general, time-frequency analysis of sensor data is completed under MATLAB, but which could not directly be inputted into the CNN. Based on this, the ST is designed and introduced into the ST-CNN as the ST layer. In the ST layer, 1D time-series sensor data samples are directly converted into time-frequency matrixes with 2D format. No manual works are needed by this way. The operation of converting the sensor data x(t) into a time-frequency matrix by the ST layer is described as:

(1) $$s\left(\tau ,f\right)=\int_{-\infty }^{+\infty }x\left(t\right)g\left(\tau -t,f\right)e^{-i2\pi ft}dt$$

where x(t) is the sensor data. f is the sensor sampling frequency. g(τ−t,f) denotes a particular normalized Gaussian window function, and its formula can be expressed as:

(2) $$g\left(t,f\right)=\frac{\left|f\right|}{\sqrt{2\pi }}e^{-\frac{t^{2}f^{2}}{2}}$$

After Equation (1), the 1D time-series sensor data such as vibration signal is converted into a time-frequency complex matrix with 2D format (i.e., s(τ,f)) and as the output of the ST layer, where the rows denote the frequencies and the columns indicate the time value. After the ST layer, the output of the ST layer is directly inputted into the CNN.

### 2.2. Convolutional Layer

For each convolutional layer, the number of filter kernel could be defined according to the need. In each convolutional layer, its kernel parameters are convolved with the data points of input. In this paper, the input of the convolutional layer is $s\left(\tau ,f\right)\in R^{A\times B}$, where A and B represents the length and width of s(τ,f) obtained from the ST layer, respectively. The output $C_{cn}$ of the convolutional layer is formulated as:

(3) $$C_{cn}=f\left(s\left(\tau ,f\right)*w_{cn}+b_{cn}\right)$$

where ∗ is the convolutional operation, $C_{cn}$ denotes cn-th feature map, cn represents the number of filter kernels, $w_{cn}$ is the weight matrix of cn-th filter kernel of the current convolutional layer and cn-th filter kernel bias is $b_{cn}$. Typically, rectified linear units (ReLU) is selected to execute f(·) in Equation (4).

### 2.3. Pooling Layer

Admittedly, the dimensionality of output feature maps will increase after the convolutional layer, and the curse of dimensionality is easily caused with the increment of the convolutional layer. Based on this, the pooling layer is used to reduce the dimensionality of output feature maps. In the pooling layer, the dimensionality of the feature maps obtained from convolutional layer is eliminated by statistical methods such as max-pooling or average-pooling. This process is expressed as:

(4) $$P_{cn}=f\left(\beta down\left(C_{cn}\right)+b\right)$$

where β is the multiplicative bias term, $C_{cn}$ is the inputs, $down\left(C_{cn}\right)$ denotes the pooling operation, b is the additive bias vector, and f(·) is the activation operation.

### 2.4. Fully-Connected Layer

Like traditional neural network, multiple neurons of fully-connected layer are used to non-linearly fit its input. All neurons are connected to all data point of feature maps from the last pooling layer such as MP 3. Its operation process is described as:

(5) $$F\left(P_{L}\right)=f\left(wP+b\right)$$

where P is the outputs of the last pooling layer, $F\left(P_{L}\right)$ represents the outputs of current fully-connected layer, w and b denotes the weight and additive bias term, respectively. f(·) is the activation operation.

### 2.5. Classification Layer

Softmax is commonly used in the classification layer, which is the generalization of the logistic classifier for solving the multi-classification problem [38]. Sensor data sample *x* output *f* through above layers, and its predicted category is determined by p(y=j|f). For the classification layer, its output is a vector with k-dimension, and the sum of the values of each element in this vector is 1. Its mathematical formula is given as:

(6) $$h_{\gamma^{T}}\left(f^{\left(i\right)}\right)=\left[\begin{array}{c} p\left(y^{\left(i\right)}=1|f^{\left(i\right)};\gamma_{1}^{T}\right)\\ p\left(y^{\left(i\right)}=2|f^{\left(i\right)};\gamma_{2}^{T}\right)\\ \vdots \\ p\left(y^{\left(i\right)}=k|f^{\left(i\right)};\gamma_{k}^{T}\right) \end{array}\right]=\frac{1}{\sum_{j=1}^{k}e^{\gamma_{j}^{T}f^{\left(i\right)}}}\left[\begin{array}{c} e^{\gamma_{1}^{T}f^{\left(i\right)}}\\ e^{\gamma_{2}^{T}f^{\left(i\right)}}\\ \vdots \\ e^{\gamma_{k}^{T}f^{\left(i\right)}} \end{array}\right]$$

where $\gamma_{1}^{T},\;\gamma_{2}^{T},\cdots ,\;\gamma_{k}^{T}$ are the parameters of regression model and 1∑j=1keγjTf(i) is to normalize the outputs.

Then, the cost function $M\left(\gamma^{T}\right)$ is defined as:

(7) $$M\left(\gamma^{T}\right)=-\frac{1}{m}\left[\sum_{i=1}^{m}\sum_{j=1}^{k}1\left\{y^{i}=j\right\}\log\frac{e^{\gamma_{j}^{T}f^{\left(i\right)}}}{\sum_{l=1}^{k}e^{\gamma_{j}^{T}f^{\left(i\right)}}}\right]$$

where 1{·} is an indicative operation, which means that when the value of brace is true, its result is 1. Otherwise, its result is 0. The cost function is minimized by stochastic gradient descent algorithm.

## 3. Proposed Fault Diagnosis Method Based on ST-CNN

**Figure 2:**
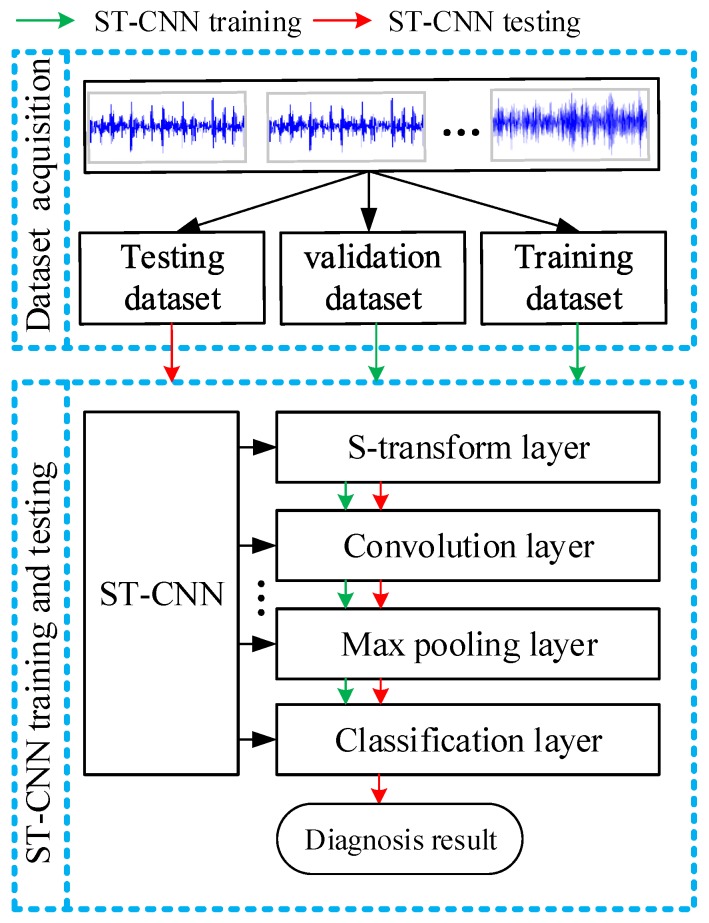
The flowchart of proposed fault diagnosis method.

In this paper, a novel fault diagnosis method is proposed based on ST-CNN. With the capability of directly extracting features from original sensor data, no manual data conversion work is needed for bearing fault diagnosis. Figure 2 shows the flowchart of the proposed bearing fault diagnosis method. In the first step, sensor data of bearings in different conditions are collected. Data samples are obtained by a sampling window from sensor data. All the obtained data samples are then randomly divided into training, validation, and testing dataset. In the second step, the training dataset is used to train the proposed ST-CNN by reducing the training error. The validation dataset is used to verify the diagnostic performance of the trained ST-CNN and prevent possible overfitting and select the trained model. The testing dataset is adopted to evaluate the generalization capability of the proposed method.

In the training process of the ST-CNN, the training samples with 1D format are directly inputted into the proposed ST-CNN, and automatically converted into the time-frequency coefficient matrix by the ST operation. The output L0 of the ST operation is used as the input $s\left(\tau ,f\right)\in R^{A\times B}$ for the first convolutional operation. After the input s(τ,f) is convolved with the filter kernel of convolutional operation, cn feature maps are obtained, which is formed as L1. The back-to-back pooling operation is used to reduce the dimensionality of L1. After the convolutional and pooling operation, the representative features are obtained from the training sample. In addition, the predicted result of the representative features could be obtained through classification stage, which is composed of one fully-connected layer (i.e., FC1) and the softmax layer (i.e., SFM). In SFM, the output neurons are transformed to the logits by Equation (6) to cater the form of probability distribution for the number of diagnosis types. Then, the training error of the ST-CNN will gradually minimize using Equation (7). After training, the ST-CNN directly extracts the representative fault features from sensor data. The fault diagnosis can be performed on new monitoring sensor data by the trained ST-CNN.

## 4. Experiment Studies

This section uses two public available bearing datasets to evaluate the effectiveness of the proposed method.

### 4.1. Case One: Bearing Fault Diagnosis with Different Defect Severity

#### 4.1.1. Experimental Setup and Data Description

**Figure 3:**
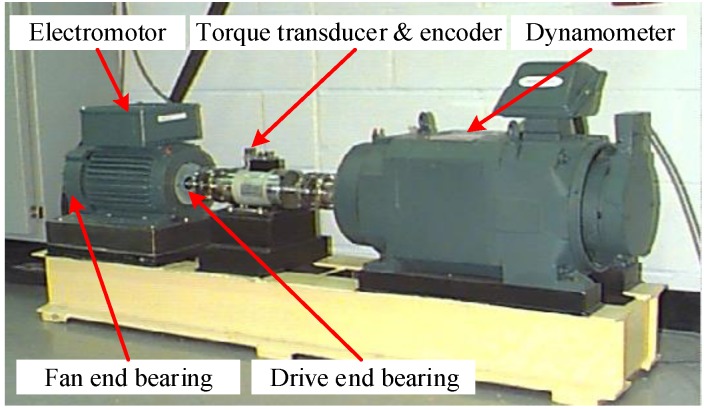
Experiment setup [39].

In this case, a public available roller bearing dataset coming from the Case Western Reserve University (CWRU) motor drive system is analyzed [39]. As shown in Figure 3, the experimental setup main includes two hp motors (left), a torque transducer/encoder (center), a dynamometer (right), and control electronics (not shown). Two accelerometers are placed at the 12 o’clock position of the drive and fan end of the motor housing. Vibration signals of bearings are collected by the two accelerometers. Different degrees of single point fault diameters are introduced at outer race, inner race and ball of bearings by using electro-discharge device.

**Figure 4:**
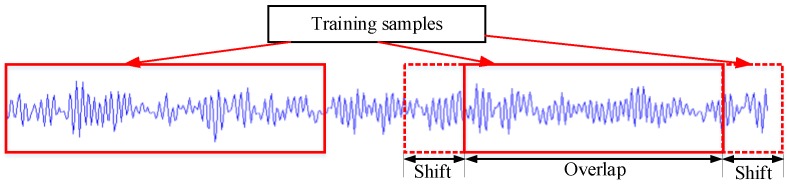
Data argument with overlap.

In this experiment, there are three types of bearing faults, which are inner ring fault (IRF), outer race fault (ORF) and ball fault (BF), as well as a normal bearing condition. Each bearing fault type contains three fault diameters: 0.007 inch, 0.014 inch, and 0.021 inch. There are three load conditions (1, 2 and 3 hp). Each load condition contains ten types of bearing dataset. In order to expand the number of training samples, a sample augmentation technique is employed in each bearing dataset. As shown in Figure 4, training samples are sliced with the window of 256 points. Then, 2000 samples are obtained from each bearing condition dataset.

In this study, there are four sets of datasets, which are dataset A, B, C and D. Dataset A, B and C correspond to three load conditions respectively, and each dataset obtains a total of 20,000 samples. Dataset D is generated by randomly selecting the data samples from the above three datasets, which consider the impact of three load condition. In each dataset, 14,000 samples are selected as training set, 3000 samples for validation set, and 3000 samples for testing set. The details of dataset D are shown in Table 1, and other datasets are similar. For each dataset, the training set is used to train the proposed ST-CNN model, and validation set is used to prevent possible overfitting and stop training process when the error rate decreases slightly or even starts to increase. Testing set is used to evaluate the performance of the proposed method.

#### 4.1.2. ST-CNN Testing Result

**Figure 5:**
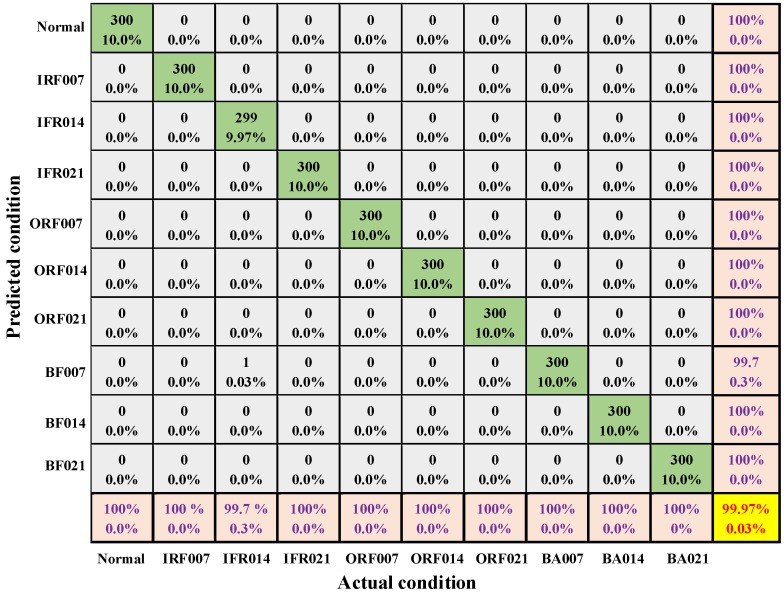
Condition classification confusion matrix in case one.

The parameters of the ST-CNN are shown in Table 2. Parameters are optimized by using mini-batch stochastic gradient descent with a batch size of 32. Table 3 shows the testing result of ten trials for the proposed method on four datasets. From the result, the proposed method is satisfactory in each dataset. In particular, the diagnosis performance on dataset D is outstanding. The mean accuracy of ten trials is 99.90%, maximum accuracy is 99.97%, minimum accuracy is 99.80%, and its standard deviation is 0.0570%. The standard deviation of ten trails shows a more reliable performance for the proposed method. Figure 5 presents the confusion matrix of the best of ten trials for dataset D. In the confusion matrix, rows stand for the actual label, and columns stand for predicted label for each condition. Seen from Figure 5, the overall diagnosis accuracy of ten condition is 99.97%, error rate is 0.03%, and ORF007, ORF014, ORF021, IRF007, IRF021, BF0.007, BF014, BF021 and Normal is 100%, while IRF014 is the worst one but its accuracy still reaches to 99.63% and it only has one error diagnosis. For the worst in the ten trials, there are overall five-error diagnosis and the fault diagnosis is satisfactory for 3000 testing samples. From Figure 5, the $F_{1}$ score [40] also could be obtained, and the mean $F_{1}$ score of all the class label is 99.97%. In addition, the mean $F_{1}$ score of all the class label of the worst trial is 99.92%.

In the proposed ST-CNN, the size of the feature map from MP 3 has a great impact on the fully-connected layer and classification layer. The output size of these feature maps could be changed by modifying the stride of convolutional layer or pooling layer. The result of ten trials of these ST-CNNs with different output sizes is presented in Table 4. In these ST-CNNs, the output size 1 × 3, output size 2 × 6 and output size 6 × 14 is better than output size 1 × 2, and the mean accuracy of ten trials of output size 6 × 14 is 99.965%, the minimum mean accuracy of ten condition is 99.90%, the maximum mean accuracy of ten condition is 100%, and the standard deviation of ten trials is of 0.0299%. From the tuning result, the fault diagnosis of the proposed method is outstanding, and it is close to 100%.

#### 4.1.3. Compared with Other Methods

Three time-frequency analysis methods based on the similar CNN compared with the proposed method. Three time-frequency analysis methods, that is, STFT, HHT and continuous wavelet transform (CWT), are used as well as ST. The comparison result is presented in Table 5 after ten trials. From the comparison result, the ST-CNN performs the higher and more reliable diagnosis performance in all ten trials than the three time-frequency analysis methods integrated with the similar CNN. Furthermore, the other methods including support vector machine (SVM) [41], k-nearest neighbor (KNN) [42], bagged trees (BT) [43] and linear discriminant (LD) [44] are compared with the proposed method. The comparison result is presented in Table 6 after ten trials. From the comparison results, the proposed ST-CNN achieves the higher diagnosis performance.

In this case study, all experiment methods are performed on a computer (Intel Core (TM) 3.6 GHz processor with 8GB of RAM) and a windows version of the tensorflow platform. All mentioned methods are trained by using the same training set. The training time and testing time of mentioned methods are presented in Table 7. In this table, the training time of ST-CNN, CWT+CNN, HHT+CNN and STFT+CNN of 30 epochs and the testing time of one testing sample are calculated. The training time of SVM, KNN, BT and LD and the testing time of one testing sample are also counted, respectively. Seen from this table, the proposed ST-CNN consumes more time than STFT+CNN, SVM, KNN, BT and LD. It is because the process of converting sensor data with the 1D time series format into time-frequency complex matrix will consume some time. However, the capability of the computer has a great impact on the training and testing performance. In addition, the testing time of the proposed ST-CNN for one testing sample is only 8.1 ms, which is smaller the human reaction speed (100–400 ms). Therefore, the proposed method is suitable for real-time diagnosis of bearing.

### 4.2. Case Two: Bearing Fault Diagnosis in Different Fault and Load Conditions

#### 4.2.1. Experimental Setup and Data Description

**Figure 6:**
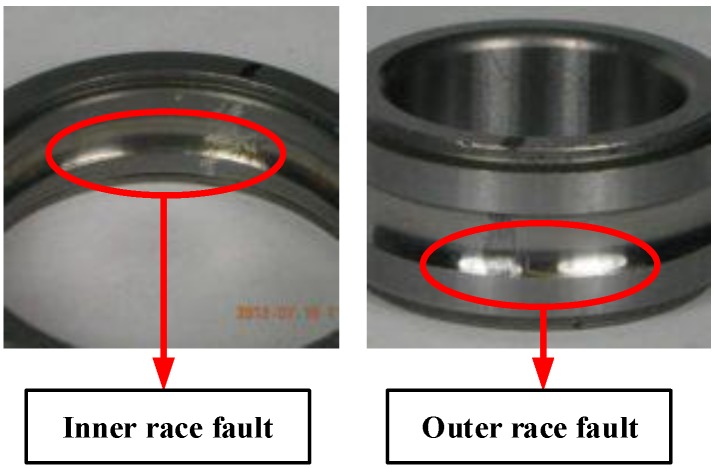
Experimental setup for bearing fault diagnosis.

In this case, a new set of bearing fault datasets from the Mechanical Failures Prevention Group (MFPT) [45] are used to evaluate the diagnosis performance of the proposed method. These fault datasets consist of monitoring signals of NICE bearings, whose roller diameter is 0.235 inch, pitch diameter is 1.245 inch, number of elements is eight and their contact angle is zero. Among these datasets, inner ring fault and outer ring fault datasets are obtained under various loads. The speed of input shaft of the test rig is 25 Hz. The sampling rate of accelerometers mounted on the bearing is 48,828 Hz. Figure 6 shows two bearings with inner race fault (IRF) and outer race fault (ORF), respectively. Two bearings fault sensor data are collected by the accelerometer.

In this experiment, there are a total of six load conditions, which are 50, 100, 150, 200, 250 and 300 lbs. The fault dataset for each load condition consists of IRF and ORF dataset. Each type of bearing fault provides 2000 samples with 256 data point. Six fault datasets are available, which are dataset A, dataset B, dataset C, dataset D, dataset E and dataset F. For each fault dataset, 2800 samples are used as training set, 600 samples as validation set, and 600 samples as testing set. Furthermore, dataset G contains 2800 training samples, 600 validation samples and 600 testing samples, which are uniformly extracted from six fault datasets. The details of these datasets are presented in Table 8.

#### 4.2.2. ST-CNN Testing Result

**Figure 7:**
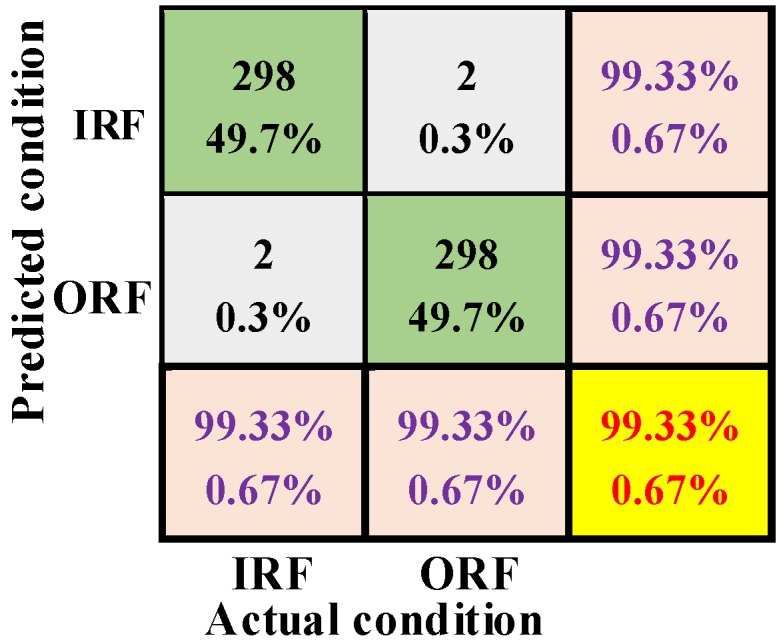
Condition classification confusion matrix in case two.

The testing result of the ST-CNN is given in Table 9. From the testing result, the diagnosis performance of the ST-CNN is satisfactory in different datasets. The mean diagnosis accuracy of ten trials is 98.80% and the standard deviation of ten trials is 0.4270 on dataset G. Figure 7 shows the confusion matrix of the best diagnosis performance of the proposed method in ten trials. Seen from the confusion matrix, only two samples of 300 samples of each failure type are diagnosed incorrectly, and its $F_{1}$ score is 99.33%. For the worst of ten trials, 11 testing samples are diagnosed incorrectly which is also satisfactory.

To improve the diagnosis performance of the ST-CNN, the size of the feature maps of MP 3 is changed by modifying the stride of the convolutional layer or pooling layer. Table 10 shows the fault diagnosis performance of different ST-CNNs. Seen from the result, output size 1 × 3, output size 2 × 6, output size 6 × 14 and output size 30 × 62 are better than output size 1 × 2. Additionally, the mean accuracy of output size 2 × 6 in ten trials is 99.50% and its standard deviation is 0.4073%.

#### 4.2.3. Compared with Other Methods

In the section, the diagnosis performance of the proposed ST-CNN is compared with other methods based on three different time-frequency analysis methods and the similar CNN. Table 11 shows the comparison result after ten trials. Seen from the result, the mean accuracy of CWT is 97.951%, HHT is 91.151%, STFT is 94.551%, and the ST-CNN preforms the higher diagnosis accuracy. In addition, the proposed method compared with other methods such as SVM, KNN, BT and LD. The comparison result is presented in Table 12. From the comparison result, the proposed method also preforms the higher performance than other methods. The training time and testing time of all experiment methods are shown in Table 13. In this table, the testing time is satisfactory.

## 5. Conclusions

In this paper, a novel sensor data-driven fault diagnosis method is proposed based on the ST-CNN. The ST is introduced into the ST-CNN as the input processing layer named ST layer. The time-frequency coefficients matrix with 2D format is obtained directly from original sensor data by the ST layer where no manual conversion work is needed. In this regard, the representative fault features are automatically extracted from original sensor data by training the constructed ST-CNN. Two public available bearing datasets are used to evaluate diagnosis performance. The testing and comparison results show that the proposed method could achieve higher and more reliable diagnosis performance for bearing faults than other existing methods.

In our future work, the fault feature extraction based on ST-CNN will be used to obtain the correlation features between multi-type faults and single faults, and the feasibility of the proposed method applied to the diagnosis of multi-type fault will be discussed.

## Figures and Tables

**Table 1:** Description of used dataset in case one.

Condition Type	Defect Severity (inch)	Dataset Division (Training/Validation/Testing)
Normal	0	1400/300/300
IRF	0.007	1400/300/300
IRF	0.014	1400/300/300
IRF	0.021	1400/300/300
ORF	0.007	1400/300/300
ORF	0.014	1400/300/300
ORF	0.021	1400/300/300
BF	0.007	1400/300/300
BF	0.014	1400/300/300
BF	0.021	1400/300/300

**Table 2:** The parameters of the constructed CNN.

No.	Layer Type	No. of Filters	Kernel Size	Stride	Output Size	Padding
1	Convolution 1	32	2 × 2	2 × 2	64 × 128	No
2	Max-pooling 1	N/A	2 × 2	2	32 × 64	No
3	Convolution 2	64	2 × 2	2 × 2	16 × 32	No
4	Max-pooling 2	N/A	2 × 2	2	8 × 16	No
5	Convolution 3	128	2 × 2	2 × 2	4 × 8	No
6	Convolution 4	256	2 × 2	2 × 2	2 × 4	No
7	Max-pooling 3	N/A	2 × 2	2	1 × 2	No

**Table 3:** Result of the ST-CNN in different load conditions in case one (%).

Dataset	A	B	C	D
Max	100	100	100	99.97
Min	99.90	99.83	99.90	99.80
Mean	99.977	99.939	99.974	99.900
Std	0.0356	0.0642	0.0347	0.0570

**Table 4:** Result of the ST-CNN with the different output size (%).

Output Size	1 × 2	1 × 3	2 × 6	6 × 14	14 × 30	30 × 62
Max	99.97	99.97	100	100	100	100
Min	99.80	99.87	99.70	99.90	99.83	99.73
Mean	99.900	99.917	99.924	99.965	99.887	99.869
Std	0.0570	0.0356	0.1201	0.0299	0.0546	0.1026

**Table 5:** Comparison of bearing fault diagnosis using other time-frequency analysis methods (%).

Methods	ST	CWT	HHT	STFT
Max	100	99.17	98.83	97.50
Min	99.90	98.87	98.23	95.83
Mean	99.965	99.000	98.486	96.982
Std	0.0299	0.1141	0.1872	0.4447

**Table 6:** Comparison of bearing fault diagnosis result using different methods (%).

Methods	Mean Accuracy
ST-CNN	99.96
SVM	94.65
KNN	98.65
BT	71.70
LD	79.80

**Table 7:** Cost time for the proposed method and other method.

Methods	Training Time (s)	Testing Time (ms)
ST-CNN	4860.1	81
CWT + CNN	10981.4	179
HHT + CNN	18293.8	495
STFT + CNN	2902.9	24.6
SVM	256.7	1.0
KNN	85.1	1.3
BT	606.1	0.08
LD	8.76	0.05

**Table 8:** Description of the dataset in case two.

Fault Type	Dataset Division (Training/Validation/Testing)						
	Dataset A	Dataset B	Dataset C	Dataset D	Dataset E	Dataset F	Dataset G
IRF	1400/300/300	1400/300/300	1400/300/300	1400/300/300	1400/300/300	1400/300/300	1400/300/300
ORF	1400/300/300	1400/300/300	1400/300/300	1400/300/300	1400/300/300	1400/300/300	1400/300/300

**Table 9:** Result of the ST-CNN in different load conditions in case two (%).

Dataset	A	B	C	D	E	F	G
Max	99.67	99.83	99.50	97.67	98.17	98.33	99.33
Min	99.50	98.83	98.67	97.17	97.00	96.67	98.17
Mean	99.584	99.647	99.231	97.485	97.548	97.684	98.80
Std	0.1425	0.3373	0.2617	0.2000	0.4113	0.5047	0.4270

**Table 10:** Result of the ST-CNN with different output size (%).

Output Size	1 × 2	1 × 3	2 × 6	6 × 14	14 × 30	30 × 62
Max	99.33	99.50	99.83	99.33	99.83	99.00
Min	98.17	99.17	98.50	99.00	99.00	98.50
Mean	98.80	99.349	99.500	99.249	99.424	98.783
Std	0.4270	0.1222	0.4073	0.1155	0.2220	0.1925

**Table 11:** Comparison of bearing fault diagnosis using other time-frequency analysis methods (%).

Methods	ST	CWT	HHT	STFT
Max	99.83	98.33	92.33	95.50
Min	98.50	97.50	90.17	94.00
Mean	99.500	97.951	91.151	94.551
Std	0.4073	0.2604	0.7704	0.4373

**Table 12:** Comparison of bearing fault diagnosis result using different methods (%).

Methods	Mean Accuracy
ST-CNN	99.50
SVM	93.60
KNN	99.00
BT	93.30
LD	59.40

**Table 13:** Cost time for the proposed method and other method.

Methods	Training Time (s)	Testing Time (ms)
ST-CNN	1060.2	27
CWT + CNN	2439.2	35
HHT + CNN	683.1	6.7
STFT + CNN	3634.4	52
SVM	5.8	0.053
KNN	3.7	0.26
BT	10.6	0.091
LD	1.9	0.032
